# Efficacy of high doses of ivermectin–clorsulon in cattle on *Glossina palpalis gambiensis* survival and fecundity: implications for human and animal trypanosomoses control

**DOI:** 10.1186/s13071-026-07277-5

**Published:** 2026-02-06

**Authors:** Sié Hermann Pooda, Soumaïla Pagabeleguem, Ange Irénée Toé, Adrien Marie Gaston Belem, Karine Mouline, Philippe Solano

**Affiliations:** 1 Institut des Sciences de l’Environnement et du Développement Rural (ISEDR), Université Daniel Ouezzin COULIBALY (UDOC), Dédougou, Burkina Faso; 2 Centre International de Recherche Développement sur L’élevage en Zone Subhumide (CIRDES), Bobo-Dioulasso, Burkina Faso; 3 Direction de L’Entomologie Et de La Lutte Contre Les Maladies Animales À Vecteurs (DELMA), Bobo-Dioulasso, Burkina Faso; 4 Centre Universitaire de Tenkodogo, Tenkodogo, Burkina Faso; 5 Université Nazi Boni, Bobo-Dioulasso, Burkina Faso; 6 MIVEGEC, Université de Montpellier, IRD, Montpellier, France; 7 INTERTRYP, Université de Montpellier, CIRAD, IRD, Montpellier, France

**Keywords:** Ivermectin, Trypanosomes transmission, Vector control, Vectorial capacity, Integrated control, Cattle treatment, *Glossina palpalis gambiensis*, Tsetse fly, Human and animal trypanosomoses

## Abstract

**Background:**

Trypanosomoses are parasitic diseases caused by *Trypanosoma* protozoa transmitted by tsetse flies (*Glossina* spp.) to humans and animals. These diseases cause major health and economic disruptions in sub-Saharan Africa. Despite the development and wide implementation of control strategies, the disease burden remains high and complementary tools are needed. Ivermectin is an endectocide toxic to arthropods, including *Glossina*. The aim of this study was to test the efficacy of different doses of ivermectin administered to cattle on the survival and fecundity of *Glossina palpalis gambiensis* Vanderplank, 1949 in Burkina Faso.

**Methods:**

This study compared the survival and fecundity of tsetse flies exposed to cattle treated with ivermectin–clorsulon (onefold veterinary therapeutic dose [TD; 0.2 mg/kg], twofold TD [2TD; 0.4 mg/kg], and fourfold TD [4TD; 0.8 mg/kg]) with those of flies exposed to control cattle (no treatment). Direct-skin blood-feeding experiments were performed at different days post-injection (DPI) (DPI: 1, 8, 15, 22, 29, and 36). The 30-day fly survival was analyzed using Kaplan–Meier curves and Cox proportional hazards models. Fecundity parameters were compared among treatments using generalized linear modeling (GLM). Time to first pupation was also measured.

**Results:**

Fly mortality differed significantly between treatments (*χ*^2^ = 353.63, *df* = 3, *P* < 0.001), with 30-day mortality rates at 1 DPI of 24.0%, 59.8%, 88.9%, and 90.4% in the control, TD, 2TD and 4TD groups, respectively. Treatments also significantly affected pupal production (*χ*^2^ = 353.63, *df* = 3, *P* < 0.001), with a decrease of 43.6–100% relative to control at 1 DPI. In addition, in tsetse flies exposed to the treatment, the deposition of the first larva occurred 9–10 days later than in nonexposed flies, in both the 2TD and 4TD groups at 1 DPI. In the 4TD group, toxic effects lasted until 15 DPI (survival) and 8 DPI (fecundity parameters).

**Conclusions:**

In our experiment, blood meals from cattle treated with an ivermectin–clorsulon formulation significantly reduced tsetse fly survival and fecundity, two key traits influencing vectorial capacity. Thus, treatment of domestic animals with the formulation has the potential to reduce trypanosomes transmission and improve both human and animal health in sub-Saharan Africa.

**Graphical Abstract:**

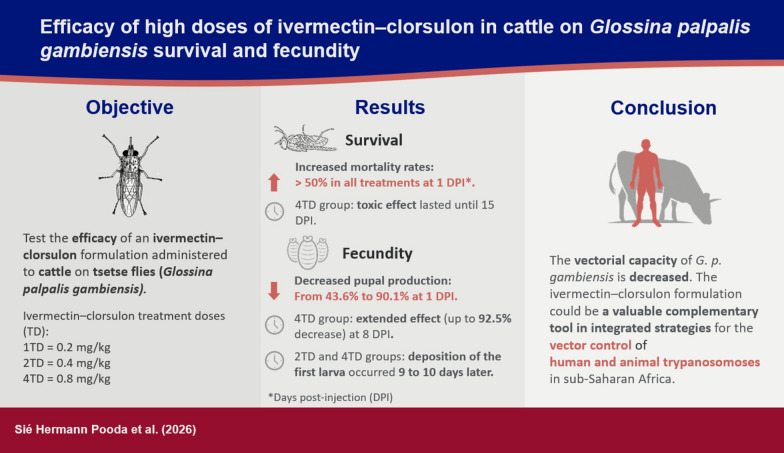

## Background

Livestock production plays a critical role in the economies of developing countries, contributing to approximately 10–20% of gross domestic product [1]. Cattle are particularly valuable due to their multifaceted contributions, including meat and milk production, draft power, and manure for use as fertilizer. Parasitic infections are a major constraint on livestock production, leading to reduced productivity, increased mortality, and higher management costs [2, 3]. African animal trypanosomosis (AAT), also known as nagana, is one of the most significant parasitic diseases limiting livestock development in sub-Saharan Africa [4, 5]. The disease is caused by hemoparasites of the genus *Trypanosoma*, which are primarily transmitted to animals by tsetse flies (Diptera: Glossinidae). These vectors are endemic in Africa and also transmit *Trypanosoma* species responsible for human African trypanosomiasis (HAT), or sleeping sickness. Together, HAT and AAT cause substantial health, social, and economic burdens across the African continent [6]. Control of HAT relies mainly on active medical screening and treatment of confirmed cases, complemented by interventions targeting tsetse fly populations. Chemotherapy remains the main strategy for controlling AAT, despite the increasing emergence of drug-resistance in trypanosomes [7, 8]. In the absence of effective vaccines for either disease, vector control remains a key preventive measure [9]. Indeed, vector control interventions have led to substantial reductions in tsetse fly populations, and consequently, in the transmission of both HAT and AAT [4, 10, 11]. The main methods for tsetse fly control include insecticide-impregnated traps and targets, topical application of insecticides to cattle acting as live bait, ground and aerial insecticide spraying, and the sterile insect technique (SIT) [12].

Although effective, current tsetse fly control methods present significant operational and economic limitations. Toxic attractants are costly to maintain: fabrics and insecticides require regular replacement, demand substantial human resources, and show reduced efficacy at low tsetse densities. Their fixed placement also limits coverage in environments where tsetse populations fluctuate with biotope, season, climatic conditions, and environmental events such as floods, bushfires, or vegetation growth. Sustainability is further challenged by limited community engagement, leading to damage or theft of traps and targets. Ground and aerial insecticide spraying can be effective under certain conditions [13–15]. However, these approaches are expensive and largely restricted to areas with gentle terrain and open vegetation. In contrast, topical treatment of livestock, informed by knowledge of tsetse biting behavior and preferential feeding sites, enables targeted insecticide application to specific body areas. This approach reduces insecticide use, operational costs, and environmental impact [16–19], with additional savings achievable through collective implementation by farmers [20]. The SIT method, while technically feasible, remains costly and is generally applied only under specific ecological and operational conditions, typically after substantial reduction of tsetse populations using other control strategies [21].

Despite substantial control efforts, tsetse flies and trypanosomosis transmission continue to impose major public health and economic burdens in affected regions. There is therefore a need to explore complementary tools for controlling both AAT and HAT, particularly in the context of current disease elimination goals [22]. One promising option is the use of ivermectin, a broad-spectrum endectocide widely used in both human and veterinary medicine.

Ivermectin belongs to the avermectin family of macrocyclic lactones, which was discovered in 1973 [23]. These compounds, referred to as endectocides, are effective against both internal (endo-) and external (ecto-) parasites [23, 24]. In domestic animals, ivermectin is widely used against a broad range of parasites, including gastrointestinal nematodes (roundworms), extraintestinal nematodes such as *Dictyocaulus viviparus* and *Parafilaria bovicola*, microfilariae of the *Onchocerca* genus, as well as various arthropods (insects and mites) [24, 25]. Surveys among livestock farmers in Burkina Faso show that ivermectin is one of the most commonly used anthelmintics for livestock deworming [26].

The standard therapeutic dose (TD) for field treatment in cattle is 0.2 mg ivermectin/kg body weight [27]. Following administration, ivermectin circulates systemically and can adversely affect blood-feeding insects, including tsetse flies, by disrupting key life-history traits. The systemic insecticidal effect of ivermectin on adult *Glossina palpalis palpalis* was first reported by Distelmans et al. [28], who observed mortality in tsetse flies fed on goats treated with a high dose of 10 mg/kg (50 times the TD), and on guinea pigs treated with 2 mg/kg (10 times the TD) [28]. Mortality typically occurred 4–8 days after repeated blood meals. These authors further reported that the onset of ivermectin-induced effects in *G. palpalis palpalis* occured at doses ranging from 1 to 2 mg/kg body weight. Subsequent studies confirmed similar systemic effects in *G. morsitans* [29], *G. tachinoides* [30], and notably, *G. palpalis gambiensis*, a major vector of both HAT and AAT in West Africa [31]. In the latter study, ivermectin administered at the standard TD of 0.2 mg/kg produced only a short-term insecticidal effect on *G. palpalis gambiensis*, lasting less than 1 week post-treatment [31].

These findings highlight the need to investigate alternative dosing regimens capable of sustaining systemic insecticidal activity over longer periods to achieve meaningful reductions in tsetse fly populations and trypanosomes transmission. Therefore, the present study aimed to identify efficacious and optimized doses of ivermectin–clorsulon on key life-history traits of *G. palpalis gambiensis*.

## Methods

### Tsetse fly species and rearing conditions

A reference colony of *G. palpalis gambiensis* maintained at the Centre International de recherche-développement sur l’élevage en zone subhumide (CIRDES) was used in this study. The colony was established in 1972 from specimens collected at Guinguette, a locality near Bobo-Dioulasso, Burkina Faso [32–34]. Since its establishment, the colony has been maintained in a dedicated insectary under controlled conditions (25 ± 1 °C and 70 ± 5% relative humidity) and fed on cattle blood provided through silicone membranes.

Newly emerged flies were sexed and placed into Roubaud cages (13.5 × 8 × 4.5 cm), with 20 females or 20 males per cage. These were maintained under the same environmental conditions in a holding room for 2 days, corresponding to the period required for sexual maturation [35]. The 3-day-old males and females of *G. palpalis gambiensis* were then exposed to different doses of ivermectin–closurlon via treated or control animal blood meals. Females were subsequently mated with 6-day-old virgin males at a ratio of 1:3 to assess the effects of treatment on fly fecundity and fertility [36].

### Feeding and host care

Eight calves (crossbreeds between the local Baoulé and the Fulani Zebu strains), with a mean age of 3 years and a mean body weight of 120.75 ± 16.44 kg, were acquired and used as blood-meal hosts for tsetse flies. Prior to the experiment, the calves were washed with soapy water to remove any residues of previously applied antiparasitic treatments. They were subsequently dewormed with albendazole and treated with a curative trypanocide (Berenil®) to eliminate potential AAT infections.

The calves were fed with a diet consisting of rice straw and cottonseed cake, with water provided ad libitum. They were housed in stables at CIRDES protected with mesh to prevent bites from other hematophagous insects. Animal health and welfare were monitored daily by a trained veterinary technician, and antibiotic treatment was administered when clinically indicated.

### Product and cattle treatment

A subcutaneous veterinary ivermectin formulation, IVOMEC-D® (Boehringer Ingelheim, Lyon, France), was used for animal treatments. The formulation contains 1% ivermectin and 10% clorsulon as active ingredients. In cattle and sheep, ivermectin primarily targets gastrointestinal nematodes, strongyles, lungworms, and external parasites, whereas clorsulon specifically targets liver flukes. Because clorsulon alone or in combination with ivermectin was shown to have no effect on *Anopheles* survival in a study by Hongsuwong et al. [37], the effects observed in the present study are considered to be primarily attributable to ivermectin, despite the lack of data specifically assessing clorsulon effects on tsetse flies.

Subcutaneous doses of ivermectin–clorsulon were administered to cattle in the neck region at one therapeutic dose (TD; 0.2 mg/kg), two therapeutic doses (2TD; 0.4 mg/kg), and four therapeutic doses 4TD; (0.8 mg/kg) of ivermectin (Table 1). Control calves received no injection and were maintained under conditions identical to those of treated animals.

**Table 1:** Description of the experimental design of the study

	Value
Number of cattle by dose	2
Ivermectin doses tested (mg/kg)	0, 0.2, 0.4, and 0.8
Days post-injection of the ivermectin–clorsulon formulation	1, 8, 15, 22, 29, and 36
Number of blood meals taken	1
Parameters evaluated	Daily mortality, pupal production, time to first pupae, and emergence rate

### Survival follow-up

To assess the systemic insecticidal effects of ivermectin formulation on tsetse fly survival, two cages, each containing either male or female flies, were placed on the flanks of each animal for 15 min. The cages were secured with rubber bands and covered with black cloth to provide darkness and facilitate blood-feeding. After feeding on cattle, fully engorged flies were monitored daily for 30 days to record mortality. This procedure was repeated at different DPI using different batches of tsetse flies (Table 1).

Because tsetse flies are obligate hematophagous insects, subsequent blood meals were provided every 2 days using a silicone membrane feeding system, as previously described [38, 39]. Defibrinated and sterilized bovine or porcine blood obtained from the Bobo-Dioulasso slaughterhouse was used for feeding.

### Systemic effects on tsetse fly fecundity

Mating cages were placed in individual larviposition cups, and the pupae were collected daily and classified as normal or aborted third-instar larvae (L3). Normal pupae were transferred to an incubation room under controlled conditions (25 ± 1 °C and 70 ± 5% relative humidity) for adult emergence. Pupal production was recorded daily for each treatment group and cage, and adult emergence was monitored until 40 days after the last pupae were produced.

To assess the systemic effects of the ivermectin formulation on tsetse fly fecundity, several reproductive parameters were measured for each group: (i) the average number of pupae produced per blood-fed females over a 30-day period following the first blood meal; (ii) the mean time to first larva production, defined as the interval time between female emergence and the production of the first larvae; and (iii) the mean adult emergence rate from the pupae.

### Data analysis

Statistical analyses and data visualization were performed using R software [40]. Fly survival was analyzed using Kaplan–Meier survival analysis, and survival curves were compared with Cox proportional hazards models (Coxph) [41]. In these models, treatment, time post-injection, and sex were included as explanatory variables, with survival as the response variable. Multiple comparisons of survival outcomes among treatment groups were performed using the glht function from the multcomp package [42]. Cumulative mortality over the 30-day follow-up period was also calculated and analyzed to facilitate comparison among treatments.

Differences in the average number of pupae produced and in the adult emergence rates among treatments were analyzed using generalized linear models with a quasi-Poisson distribution [43]. Differences in the timing of first pupal production among treatment groups were assessed using the Kruskal–Wallis test [43].

## Results

### Effects of the ivermectin–clorsulon formulation on the survival of tsetse flies

**Figure 1:**
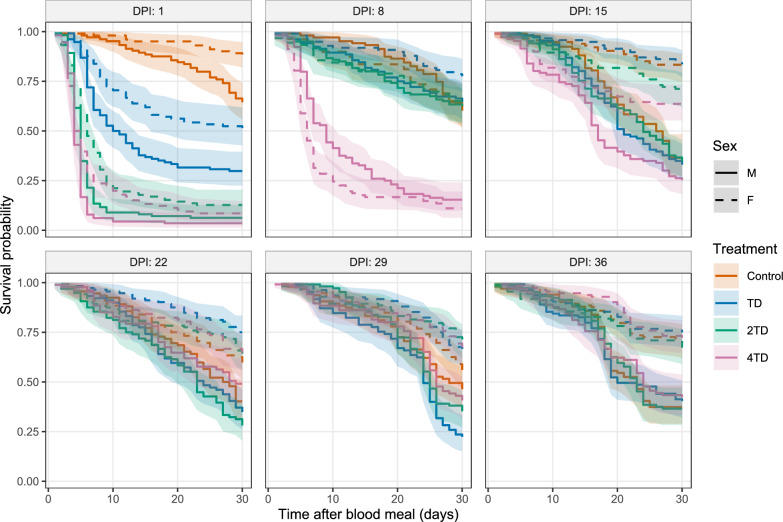
Survival curves of *Glossina palpalis gambiensis* fed on cattle treated with an ivermectin–clorsulon formulation or untreated controls. *TD* therapeutic dose of ivermectin (0.2 mg/kg), *2TD* twofold therapeutic dose of ivermectin, *4TD* fourfold therapeutic dose of ivermectin, *DPI* days post-injection of the ivermectin–clorsulon formulation, *M* male, *F* female

A total of 5001 tsetse flies (2417 males and 2584 females) were included in the survival analysis. Median survival for female flies exceeded 30 days in both the control and TD groups (Fig. 1). In contrast, median survival for control males fell below 30 days at several timepoints: 26 days (95% CI: 21-28) at 15 days post-injection (DPI), 27.5 days (95% CI: 24- > 30) at 22 DPI, 28 days (95% CI: 25- > 30) at 29 DPI, and 23 days (95% CI: 19-25) at 36 DPI. A similar pattern was observed in males from the TD group, whose survival was significantly lower than that of females. Indeed, median survival in the TD group was 24 days (95% CI: 21-25) for males and more than 30 days for females. By contrast, median survival was markedly reduced in both sexes at higher ivermectin doses, with the 2TD group reaching 27 days (95% CI: 25-30) and the 4TD group 21 days (95% CI: 19-23).

**Figure 2:**
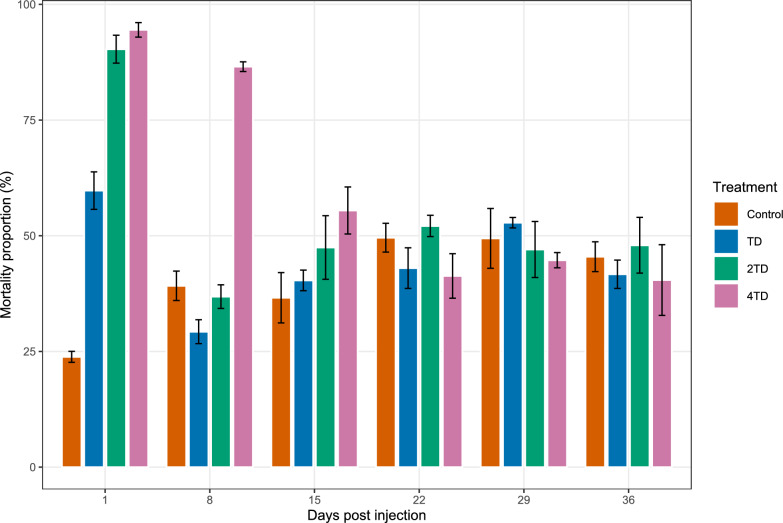
Cumulative 30-day mortality of* Glossina palpalis gambiensis* fed on cattle treated with an ivermectin–clorsulon formulation or untreated controls, at different days post-injection (DPI). *TD* therapeutic dose of ivermectin (0.2 mg/kg), *2TD* twofold therapeutic dose of ivermectin, *4TD* fourfold therapeutic dose of ivermectin

The effect of ivermectin–clorsulon formulation on *G. palpalis gambiensis* survival varied according to treatment (*χ*^2^ = 353.63, *df* = 3, *P* < 0.001), DPI (*χ*^2^ = 450.90, *df* = 2, *P* < 0.001), and fly sex (*χ*^2^ = 278.21, *df* = 2, *P* < 0.001). The TD treatment significantly reduced tsetse survival only at 1 day post-injection (1 DPI) (estimate = 1.23, *Z* = 7.36; *P* < 0.001). At this timepoint, mortality in the TD group reached 59.8% (48.6% in females and 70.2% in males), compared with 29.9% in the control group (11.9% in females and 35.6% in males) (Fig. 2). The effect of the TD treatment was significant for both females (estimate = 1.64; *Z* = 5.12; *P* < 0.001) and males (estimate = 1.09; Z = 5.47; *P* < 0.001).

The 0.4 mg /kg dose (2TD) also significantly reduced tsetse fly survival at 1 DPI only (estimate = 2.76; *Z* = −14.68; *P* < 0.001). Mortality at this time point reached 90.4% in treated flies (87.3% in females and 93.8% in males), compared with 23.9% in control flies (11.9% in females and 35.6% in males).

The highest dose (4TD) resulted in a significant reduction in tsetse fly survival at multiple timepoints: at 1 DPI (estimate = 2.76; *Z* = 16.96; *P* < 0.0001), 8 DPI (estimate = 1.72; *Z* = 11.99; *P* < 0.0001), and 15 DPI (estimate = 0.64; *Z* = 4.37; *P* < 0.001). Corresponding mortality rates were 94.6%, 86.4%, and 55.1%, at 1, 8, and 15 DPI, respectively. No significant differences in survival were observed between treated and control flies at 22, 29, and 36 DPI (*P* > 0.05). Direct comparison between the 4TD and TD groups revealed a significantly higher mortality in the 4TD group at 1 DPI (estimate = 1.52, *Z* = 13.31, *P* < 0.0001), 8 DPI (estimate = 2.01, *Z* = 13.11, *P* < 0.0001), and 15 DPI (estimate = 0.53, *Z* = 3.81, *P* < 0.001).

### Systemic effects of ivermectin formulation on fecundity

#### Mean number of pupae produced per female

**Figure 3:**
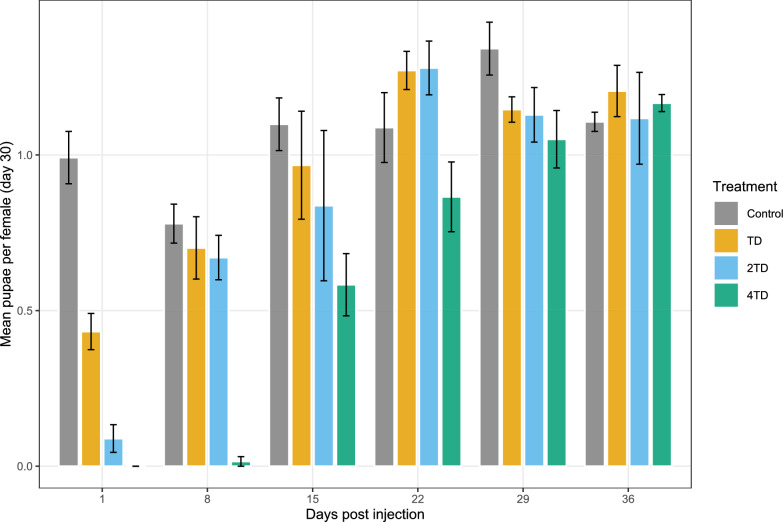
Average number of pupae produced by *Glossina palpalis gambiensis* females. *TD* therapeutic dose of ivermectin (0.2 mg/kg), *2TD* twofold therapeutic dose of ivermectin, *4TD* fourfold therapeutic dose of ivermectin, *DPI* days post-injection of the ivermectin–clorsulon formulation

Fecundity of *G. palpalis gambiensis*, expressed as the mean number of pupae produced per female, varied according to treatment (deviance = 3.41, *df* = 3, *P* < 0.001) and DPI (deviance = 15.37, *df* = 5, *P* < 0.001) (Fig. 3). Females fed on cattle treated with one TD produced an average of 0.43 ± 0.08 pupae per female at 1 DPI compared with 0.99 ± 0.06 pupae in the control group, corresponding to a significant reduction of 56.6% (estimate = −0.83, *Z* = −5.77, *P* < 0.0001). Females fed on cattle treated with 2TD exhibited an even greater reduction in fecundity at 1 DPI, producing only 0.09 ± 0.04 pupae per female, representing a 90.1% decrease relative to controls (estimate = −2.41, *Z* = −5.75, *P* < 0.0001).

The fourfold treatment dose also significantly reduced fecundity, with treated females failing to produce any pupae at 1 DPI. At 8 DPI, tsetse flies produced an average of 0.02 ± 0.02 pupae per female compared with 0.78 ± 0.06 in the control group, corresponding to a 92.5% reduction (estimate = −3.92, *Z* value = −3.38, *P* = 0.003). For all other DPI and dose combinations in which pupal production occurred, no significant differences in fecundity were observed between treated and control groups.

#### First larval period

The duration of the first larval period ranged from 14.5 to 30 days, depending on treatment and DPI (Table 2). The TD of the ivermectin formulation had no significant effect on the mean time to first larviposition. In contrast, the 2TD treatment induced a significant delay of nine days at 1 DPI (*Z* = 2.92, *P* = 0.01). Female flies fed on cattle treated with the 4TD formulation exhibited a 10-day delay in first larviposition at 8 DPI (*Z* = 3.45, *P* = 0.0017).

**Table 2:** Average number of days for first larviposition

DPI	Average number of days for first larviposition according to treatment (SE)			
	Control	TD	2TD	4TD
1	17.00 (1.05)^a^	20.71 (0.36)^a^	26.67 (1.67)^b^	30.00 (0.00)^b^
8	19.13 (0.55)^a^	20.42 (0.95)^a^	19.75 (0.63)^ab^	29.40 (0.60)^b^
15	17.50 (0.50)^ab^	17.75 (0.25)^b^	19.75 (1.55)^ab^	19.75 (0.86)^a^
22	17.25 (0.41)^a^	16.88 (0.23)^a^	17.38 (0.26)^a^	18.25 (0.37)^a^
29	17.00 (0.31)^a^	17.25 (0.25)^a^	17.50 (0.71)^a^	17.63 (0.42)^a^
36	18.00 (0.00)^a^	18.00 (0.00)^a^	18.13 (0.13)^a^	17.13 (1.03)^a^

The data in the same row with the different letters are significantly different at *P* < 0.05

*DPI* days post-injection of the ivermectin–clorsulon formulation, *TD* therapeutic dose of ivermectin, *2TD* twofold therapeutic dose of ivermectin, *4TD* fourfold therapeutic dose of ivermectin, *SE* standard error

#### Adult emergence

**Fig. 4:**
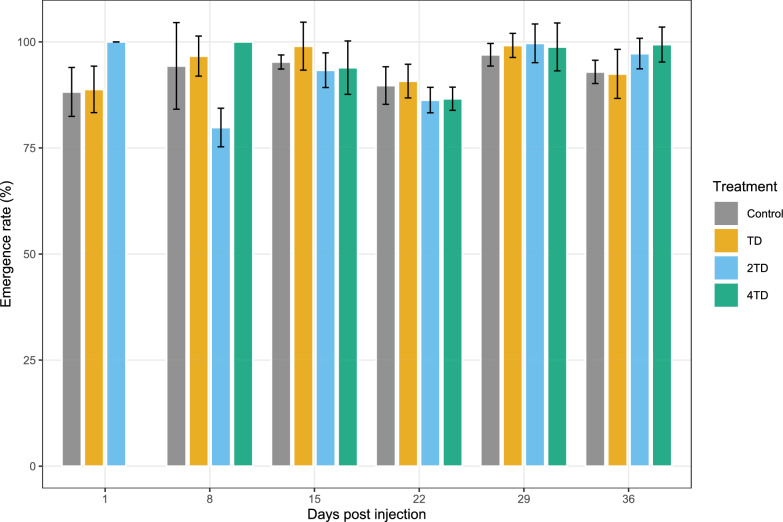
Average emergence rate of pupae produced by *Glossina palpalis gambiensis* females. *TD* therapeutic dose (0.2 mg/kg), *2TD* twofold therapeutic dose, *4TD* fourfold therapeutic dose, *DPI* days post-injection of the ivermectin–clorsulon formulation

Emergence rates of pupae produced by *G. palpalis gambiensis* females are shown in Fig. 4. No significant differences in pupal emergence were observed between control and treated groups across all doses and DPI (treatment effect: deviance = 1.46, *df* = 3, *P* = 0.86; DPI effect: deviance = 22.08, *df* = 5, *P* = 0.06).

## Discussion

This study demonstrated that treating cattle with an ivermectin–clorsulon formulation significantly reduces the survival of *G. palpalis gambiensis* feeding on treated hosts. With the therapeutic dose (TD), the insecticidal effect persisted for up to 1 day post-injection, whereas the highest dose (4TD) extended this effect to nearly 15 days post-injection. In contrast, doubling the TD did not prolong the insecticidal effect beyond 1 DPI. These findings differ from those of our previous study using the same formulation, in which the TD induced an insecticidal effect lasting up to 8 days post-treatment in the same species [31]. This discrepancy may reflect interindividual variability in ivermectin pharmacokinetics, particularly as the earlier study evaluated each dose in a single animal.

As the formulation is progressively eliminated from the bloodstream of treated cattle, variability in tsetse fly survival became apparent, especially between sexes, with females generally exhibiting greater longevity than males. Similar sex-based differences in survival have been reported previously for this species under varying temperature conditions and feeding regimes [44]. Nonetheless, careful dose selection remains essential to avoid unintended effects on female tsetse flies, particularly regarding reproductive parameters. Sublethal exposure that reduces fecundity without causing complete mortality may inadvertently increase selection pressure, potentially promoting the development of resistance over time [45].

The mean elimination half-life of ivermectin following subcutaneous administration of the commercial formulation at the TD in cattle is approximately 8 days [46–48]. In the present study, we observed that a relatively low and apparently safe dose of 4TD reduced tsetse fly survival for up to 15 days post-treatment. These findings contrast with those of Distelmans et al. [28], who reported a shorter duration of insecticidal effect despite administering a higher dose (2 mg/kg, 10TD). However, that study used guinea pigs and goats as blood-meal hosts rather than cattle, a factor likely influencing ivermectin pharmacokinetics, and consequently, the magnitude and duration of its systemic insecticidal activity [49]. Similarly, Van Den Abbeele et al. reported no significant effect on the survival of *G. palpalis palpalis* fed on guinea pigs treated with 0.5 mg/kg ivermectin [50], and Van Den Bossche and Geerts observed no impact on *G. tachinoides* fed on pigs treated with 1 mg/kg ivermectin [30]. Differences between these studies and our results may be explained not only by interspecific variation in ivermectin pharmacokinetics among host animal species, but also by differential susceptibility among tsetse fly species, as previously demonstrated for *Anopheles* mosquitoes [51]. Although unlikely, a potential contribution of clorsulon to the observed effects cannot be entirely excluded, as previous studies relied solely on ivermectin formulations.

As observed for survival, discrepancies among studies regarding the effects of ivermectin on tsetse fly fecundity are likely attributable to differences in tsetse fly species, blood-meal host, ivermectin pharmacokinetics, and experimental design, including formulation composition. In the present study, the TD of the ivermectin–clorsulon formulation reduced fecundity by 56.6% at 1 DPI. In contrast, Van Den Abbeele et al. [52] reported no effect of the TD of ivermectin on fecundity in *G. palpalis palpalis* fed on rabbits. Increasing the dose to 0.4 mg/kg (2TD) in our study did not prolong the duration of the fecundity-reducing effect in *G. palpalis gambiensis*.

Moreover, Van Den Abbeele et al. [50] reported that ivermectin administered at 0.5 mg/kg reduced fecundity (assessed by both pupal number and pupal mass) in *G. palpalis palpalis* fed at 1 DPI. In the present study, ivermectin exposure not only reduced pupal output, but also delayed the onset of first larviposition under both the 2TD and 4TD treatments. This delay persisted up to 8 DPI under the 4TD treatment, with first larviposition occurring at 29 days post-feeding in treated females, compared with 19 days in the control group. This timing observed in controls is consistent with previous observations by Jackson, who reported first larviposition at 19–20 days in tsetse flies maintained under controlled conditions with daily feeding [53]. By delaying first larviposition and reducing overall pupal production, the treatment of cattle with ivermectin has the potential to substantially limit tsetse population growth, thereby reducing the risk of transmission of trypanosomes and incidence of both HAT and AAT.

No significant effect of the ivermectin–clorsulon formulation on adult emergence rates was observed in the present study, consistent with previous reports [29]. However, earlier work on *G. morsitans* showed that a single blood meal taken from cattle 7 days after treatment with the TD of ivermectin resulted in a 44% reduction in fertility during the second ovarian cycle, after which fertility gradually returned to baseline levels [29]. According to Van Den Abbeele et al. [52], the adverse effects of ivermectin on tsetse fly fecundity are likely mediated through a combination of delayed ovulation, prolonged gestation duration, and disruption of pupal development.

The results of this study on *G. palpalis gambiensis* are consistent with findings from other tsetse species, supporting the conclusion that ivermectin, even when administered as a commercial formulation with transient activity, can negatively affect tsetse fly longevity and reproduction. These biological effects are likely to result in a meaningful reduction in tsetse fly vectorial capacity, and consequently, in reduced transmission of African trypanosomes, given the slow reproductive rate in tsetse flies and the relatively long extrinsic incubation period of the parasites. Specifically, *Trypanosoma vivax* requires approximately 10 days to complete development in the fly, *T. congolense* requires 12–14 days, and *T. brucei* requires 20–30 days [54, 55]. Therefore, any ivermectin-induced reduction in tsetse fly survival is expected to disrupt the transmission of all three major trypanosome species, with the strongest impact expected for *T. brucei*.

Although ivermectin primarily affects trypanosome transmission indirectly by reducing vector survival and preventing completion of parasite development, it would be valuable to investigate whether ivermectin administered to cattle may also exert direct effects on trypanosome development within the vertebrate host and/or the vector, as reported for other pathogens [56, 57]. Indeed, studies in other experimental systems, including in vitro assays, have shown that ivermectin can impair the development of several trypanosomes species and strains, including *T. brucei* [58], *T. evansi* [59], and *T. cruzi* [60].

The injectable formulation used in this study contains clorsulon. However, its specific contribution to tsetse fly mortality and fecundity was not assessed. Clorsulon is a flukicide that disrupts anaerobic glycolysis in *Fasciola hepatica* [61], and there is currently no evidence that it exerts toxic or metabolic effects on insects nor that it reaches biologically relevant concentrations against hematophagous dipterans following livestock treatment. To date, the only study that investigated clorsulon toxicity in insects—either alone or in combination with ivermectin—reported no effect on *Anopheles* survival [37]. In commercial formulations, clorsulon is co-administered with ivermectin, whose potent and well-characterized insecticidal activity is expected to dominate any effects on tsetse flies. For these reasons, we did not attempt to isolate the effect of clorsulon in this study, although targeted experiments would be required to definitively rule out any contribution.

Despite its potential for disease control, mass treatment of livestock with ivermectin–clorsulon formulations raises ecological concerns that should not be overlooked. Adverse effects of ivermectin on nontarget dung fauna and other wildlife have been documented [62–64], highlighting the need for responsible and targeted use of endectocides [65]. In addition, although the low reproductive rate of tsetse flies may limit the rapid emergence of insecticide resistance, proactive resistance-management strategies should be considered to preserve efficacy within integrated vector control programs. The mechanisms underlying the relatively lower susceptibility of tsetse flies (and other arthropods such as *Culex* or *Aedes* mosquitoes [66, 67]), to ivermectin compared with *Anopheles* mosquitoes, remain poorly understood [68]. Possible explanations include enhanced expression of detoxification processes and metabolic resistance pathways [69], as well as the production of ivermectin-insensitive glutamate-gated chloride (GluCl) channel variants through alternative splicing [70]. This research area warrants further investigation to identify potential resistance mechanisms and to inform future resistance monitoring and management strategies [45].

Finally, given that the insecticidal effects of veterinary-recommended formulations and doses do not last long, sustained reductions in trypanosomes transmission would require repeated treatments over time, representing a major logistical and economic constraint. An alternative approach could include the development and deployment of long-acting ivermectin formulations or implants, which can maintain plasma concentrations lethal to *Anopheles* mosquitoes for extended periods [51, 71, 72], and may offer similar benefits for tsetse fly control.

## Conclusions

This study demonstrated that the ivermectin–clorsulon formulation administered to cattle affects both the longevity and fecundity of *G. palpalis gambiensis*. Increasing the dose to 4TD extended the insecticidal effect up to 15 DPI. Endectocides in general, and ivermectin in particular, may therefore represent valuable complementary tools in integrated strategies for the control of African trypanosomosis, especially in areas where tsetse flies feed predominantly or opportunistically on domestic animals such as cattle. The observed effects on vector fecundity, combined with the potential for direct impacts on parasite development within vectors, warrant further investigation. Moreover, given that trypanosomiasis frequently co-occurs in sub-Saharan Africa with other vector-borne diseases, such as malaria, whose *Anopheles* vectors are highly susceptible to ivermectin, ivermectin-based interventions offer promising opportunities for integrated vector control targeting multiple diseases simultaneously. However, several important limitations must be considered before ivermectin can be widely promoted for vector control. These include potential adverse effects on nontarget organisms, the presence of drug residues in milk following treatment of dairy cattle, and the risk of resistance development associated with extensive or repeated use. Together, these factors emphasize the need for cautious, context-specific, and evidence-based evaluation of ivermectin-based interventions before their integration into large-scale vector control programs.

## Data Availability

Data supporting the main conclusions of this study are included in the manuscript.

## Abbreviations

AAT: African animal trypanosomosis
CI: Confidence interval
CIRDES: Centre International de recherche-développement sur l’élevage en zone subhumide
DPI: Days post-injection
SD: Standard deviation
TD: Therapeutic dose
HAT: Human African trypanosomiasis
